# Low-Energy Pulsed-Laser Welding as a Root Pass in a GMAW Joint: An Investigation on the Microstructure and Mechanical Properties

**DOI:** 10.3390/ma15217741

**Published:** 2022-11-03

**Authors:** Mariane Chludzinski, Rafael Eugenio dos Santos, Marta Ortega-Iguña, Cristina Churiaque, Manuel Porrúa-Lara, José María Sánchez-Amaya

**Affiliations:** 1 Department of Materials Science and Metallurgical Engineering and Inorganic Chemistry, School of Engineering, University of Cádiz, Av. la Universidad de Cádiz, 10, E-11519 Puerto Real, Cádiz, Spain; 2 Navantia S.A., S.M.E., Bahía de Cádiz Shipyard, Industrial Estate s/n, E-11519 Puerto Real, Cádiz, Spain

**Keywords:** gas metal arc welding, pulse laser welding, root pass, AH36 steel, microstructure, mechanical properties

## Abstract

Root pass is a fundamental step in multi-pass welding. In gas metal arc welding (GMAW), the weld bead qualities depend on the process parameters, filler materials, and welder abilities. This work investigates the effect of a Nd: YAG pulsed laser as a first pass to reduce the welders’ reliance on the AH36 low-alloy steel with 5.5 mm thickness. This autogenous automatable process delivers reduced thermal impact due to the concentrated high-energy source, pulse overlap, and higher penetration depth-to-power ratio than continuous lasers. The outcomes indicate that the PL as a root welding generated a small HAZ compared to the GMAW condition. In addition, the subsequent arc passes positively affected the microstructure, reducing the hardness from around 500 to 230 HV. The PL + GMAW achieved similar strength results to the GMAW, although its Charpy impact values at −50 °C were around 15% lower than the arc condition.

## 1. Introduction

Several industries around the world widely have adopted a great variety of welding techniques. Different areas have constantly invested in innovative joint technologies following the 5.0 industry revolution trends highlighted by social well-being and reduced environmental impacts [1]. In furtherance of this, the combination of the advantages of different welding technologies has allowed to meet this global demand. Advanced studies on alternative energy sources have brought disruptive processes and equipment in recent years. Among them, methods of laser beam welding (LBW) have presented characteristics to supply different industrial segments, such as aeronautical, naval, rail, automobile, and oil and gas [2,3,4]. In general, its precision and high concentrated energy source delivered by the laser beam melt a small area, creating deep and narrow joints [5]. In addition, it is possible to weld different materials and thicknesses without additional material.

Laser beam sources can be divided into two main wave modes, continuous and pulsed. In the continuous mode, laser irradiation is constantly emitted on the material during all welding processes, requiring robust equipment and elevated investments. On the other hand, the pulsed laser (PL) mode adopts an intermittent beam, which is emitted in short pulses with a predetermined duration (usually milliseconds) [6]. As a result, successive overlapped pulses generate a weld bead throughout a sequence of welding spots. Owing to the overlapping periodic thermal exposure, the fusion zone is re-heated above the melting point with a slow subsequent cooling stage. In addition, the average power involved achieves higher penetrations than in the continuous mode [7,8,9], allowing its application with small and economical apparatus. Furthermore, its accurate energy control permits delivering different pulse shapes to improve depth penetration, reduce defects, and join dissimilar materials [8,9,10,11,12]. In terms of the mechanical impact of the process in the FZ, its application on a St37 carbon steel alloy increased the hardness up to 200 HV [2]. In dual-phase steel, the values varied from 230 to 360 HV [13], while in AISI 1005 steel, the values reached 390 HV with an ultimate tensile strength of 288 MPa (94% of the base material) [14].

Another important aspect of LBW is the ability to be combined with other processes, sequentially or simultaneously. Currently, several industrial sectors have implemented LBW combined with GMAW, known as the hybrid laser-arc welding process (HLAW). GMAW uses an electric arc applied to a consumable wire, which becomes the filler metal and requires an inert shielding gas to protect the weld seam. The main advantages that encourage its use together with LBW are the wide-gap tolerance and control of bead humping defects [2,15,16,17]. Furthermore, this technique can be implemented in various materials, including steel, aluminium, and titanium [18,19,20,21,22]. The cooling rate generated by the process parameters and pre- and post-heat treatments is another important aspect. It contributes to mitigating undesirable phases (brittle martensite) in the laser fusion zone (FZ) and eliminate residual gases, which provides an improvement of the mechanical and metallurgical properties [23]. Some studies have shown an increase in the hardness in the laser FZ due to the fast cooling, reducing the toughness and generating brittle phases [24]. For this reason, the relevant standards for offshore and marine applications set a maximum limit of 350 HV [25,26,27]. In terms of mechanical performance, the strength should be equal or superior to the base material, and the impact energy absorbed should be higher than 34 J. Nevertheless, researchers have focused on simultaneous processes, indicating a lack of knowledge regarding the sequential application of these different welding technologies.

In particular, it is important to understand the influence of each method on the joint quality, especially in the use of PL welding before the arc process. Thus, the present contribution compares the application of GMAW after a PL welding root with a standard GMAW process on AH36 low-alloy steel plates with 5.5 mm thickness. It aims to diminish the thermal impact and reduce the material and the human reliance in the multi-pass welding process.

## 2. Materials and Methods

The material used in this investigation was AH36 steel plates cut in probes of 200 mm × 100 mm × 5.5 mm. The filler wire used for the GMAW process was AWS A5.36 E71T-1M (SC 420 MC Flux Cored Hyundai) with a diameter of 1.2 mm. The chemical compositions of the base material and the filler wire are shown in Table 1.

**Figure 1:**
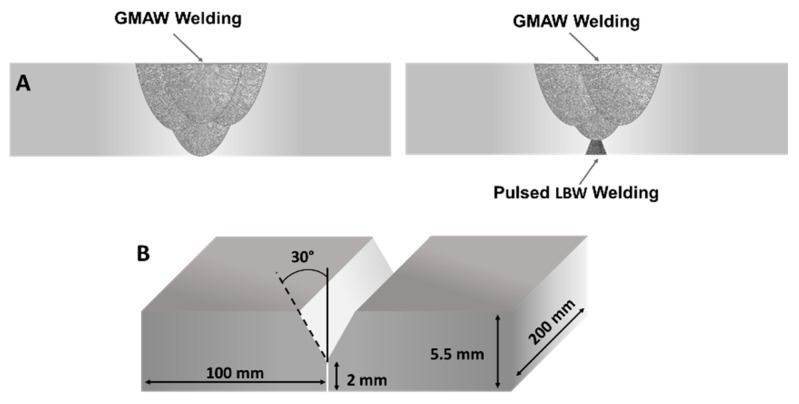
(**A**) Welding joints conditions: single-sided GMAW process (left image) and double-sided PL + GMAW process (right image). (**B**) Plate preparation with a Y-groove joint configuration.

To evaluate the influence of the root pass, the plates were butt-welded in two conditions: single-sided gas metal arc welding (GMAW) and double-sided with a combination of PL as a root treatment and subsequent GMAW welding process (PL + GMAW), as described in Figure 1A. In the GMAW samples, the arc process consisted of 4 consecutive passes with the same filling wire to reach full penetration, while in tandem PL + GMAW samples, only 3 GMAW passes were needed after the PL. Before welding, the plates were prepared in a Y-groove joint configuration, as depicted in Figure 1B.

The PL root pass was applied using Nd:YAG PL equipment using SYSMA model LM-D 100 W, with a laser wavelength of 1064 nm, maximum peak power of 9 kW, and average peak power of 80 W. Saind GZ4/E equipment was employed in the GMAW passes. The GMAW welding procedure consisted of four filling welding passes, while the PL + GMAW included the PL treatment followed by three GMAW passes. Before performing the butt joints, the PL welding trials were executed in order to determine the most adequate welding parameters to achieve at least 1.0 mm of penetration depth. The welding parameters applied in both processes are reported in Table 2.

Comparative analyses between these joint conditions were performed in terms of metallurgical and mechanical properties. The microstructure and the defects analysis were performed by metallographic standard procedures on joints cross sections. Optical microscopy (OM) was used to evaluate the welds etched with Nital 5%. Microhardness mappings were performed in a Shimadzu model HMV 2 ADW testing machine. The impact Charpy test was also performed to investigate the impact properties in a Hoytom equipment model D2M with a maximum of 300 J. Sub-sized specimens (55 mm × 10 mm × 5 mm) were extracted using an electrical discharge machining from the centre of the weld according to the ASTM E-23 standard [30]. The V-notch was located in the ZF and the values of absorbed energy were converted to standard specimens, as mentioned in DNV-OS-F101 [31]. At least three specimens from the base material and welding conditions were tested at −50 °C, in order to guarantee that all Charpy samples were separated into two pieces. A Shimadzu AGS X equipment was used in the tensile testing, where three specimens of each condition (BM, GMAW, and PL + GMAW) were tested. The dimensions of the specimens were in accordance with ASTM E8 [32], with a width of 12.5 mm. All fractured samples (Charpy and tensile testing) were observed in a Nova NanoSEM 450 FEI (Thermo Fisher Scientific – Waltham, MA, USA) scanning electron microscopy (SEM) to characterize fracture mechanisms. EDX analyses were also carried out to obtain the chemical composition.

## 3. Results and Discussion

### 3.1. Pulsed Laser Analysis

Before analysing the effect of the PL combined with the GMAW process, an evaluation of the laser process impact was performed. In this sense, a weld seam was created with the same material and dimensions used in the joints for macro- and micrographic assessments. The presence of cracks or spatter welding defects was not detected.

**Figure 2:**
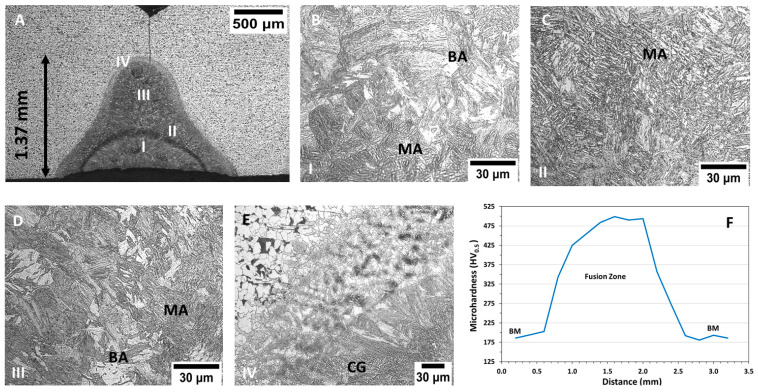
Macro- and micrographs showing the features and microstructures of the PL weld seam (**A**–**E**) and the microhardness profile (**F**). BA—Bainite, MA—Martensite, and CG—Columnar Grains.

The joint characteristics and the microstructures of the main welding areas of laser butt joint samples are shown in Figure 2. A ferritic–pearlitic matrix was noted in the BM and the weld seam displayed 1.37 mm of depth, below the 2 mm of the Y-groove height (Figure 2A). The narrow welding pool was a consequence of the highly focused laser source employed. Due to this heat input, the material was subjected to temperatures above the melting point, developing the final microstructure in the subsequent solidification stage. The effect of the pulse overlapping was observed in the centre of this fusion zone (semi-circular area in Figure 2A). During the process, each pulse partially covered the previous FZ creating a welding area superposition. For this reason, the high temperatures delivered in each pulse affected the previous region changing the microstructure due to the intermittent thermal cycles. In this sense, the FZ microstructure was mainly composed of martensite and bainite (Figure 2B,D). Columnar grains were noted on its external border, which grew perpendicular to the fusion boundary aligned at the direction of the higher heat extraction. The heat-affected zone (HAZ) exhibited around 120 μm length with its transitioned microstructure until it reached the base material (Figure 2E). Other studies carried out with steel PL weld displayed the FZ with similar characteristics concerning the pulse overlap, and the developed microstructures [14,33,34,35]. The microhardness profiles were performed at 0.5 mm from the bottom surface, and the average value measured in the BM was 190 HV. A significant rise was noted in the FZ, reaching 499 HV (Figure 2F), associated with the microstructure modifications produced by the PL. The high temperatures and cooling rates changed the ferritic–pearlitic matrix to a martensite matrix.

### 3.2. PL + GMAW and GMAW Butt Joints

#### 3.2.1. Macro- and Microstructure

**Figure 3:**
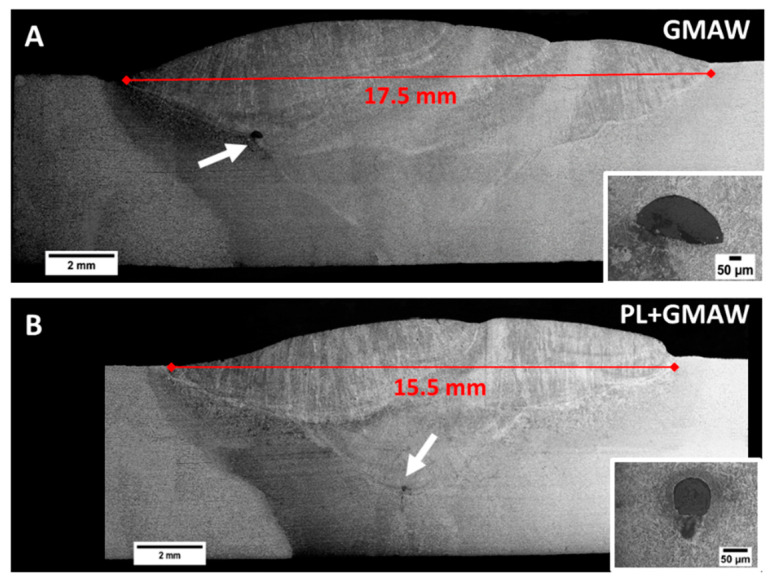
Macrographs of the weld seams made of GMAW (**A**) and PL *+* GMAW (**B**) conditions. In detail, the micrographics show the defects as indicated by the arrows.

Figure 3 shows images of the macrostructural characterization carried out in the GMAW and the PL + GMAW joints. As observed, the conditions generated full penetration joints. In addition, both weld beads showed welding inclusions in the GMAW area. In general, weld seams presented similar characteristics; however, the arc condition displayed a larger welding area. Since the PL acted as a root pass, the GMAW had one more arc pass than the PL + GMAW; therefore, this additional arc pass increased the FZ.

**Figure 4:**
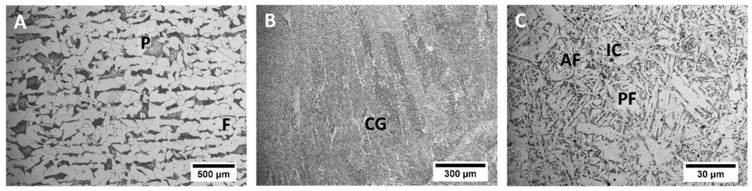
Microstructures of the base material (**A**), upper (**B**), and central (**C**) area of the last welding pass. P—Perlite, F—Ferrite, CG—Columnar grains, AF—Acicular ferrite, PF—Polygonal ferrite, and IC—Inclusion.

The microstructure characterization was performed in both GMAW and PL + GMAW weld seams. The AH36 steel base material showed a ferrite matrix with pearlite banding orientation according to the lamination manufacturing process (Figure 4A). As the PL was applied on the bottom part of the PL + GMAW joints, the upper part of both joints corresponds to the GMAW process area. This area displayed columnar grains of the last welding pass, which developed Widmanstatten, acicular, polygonal, and grain boundary ferrites microstructures (Figure 4B). The central part of this last pass generated a fine structure with bainite, acicular ferrite, polygonal ferrite, and some dispersed inclusions (Figure 4C).

**Figure 5:**
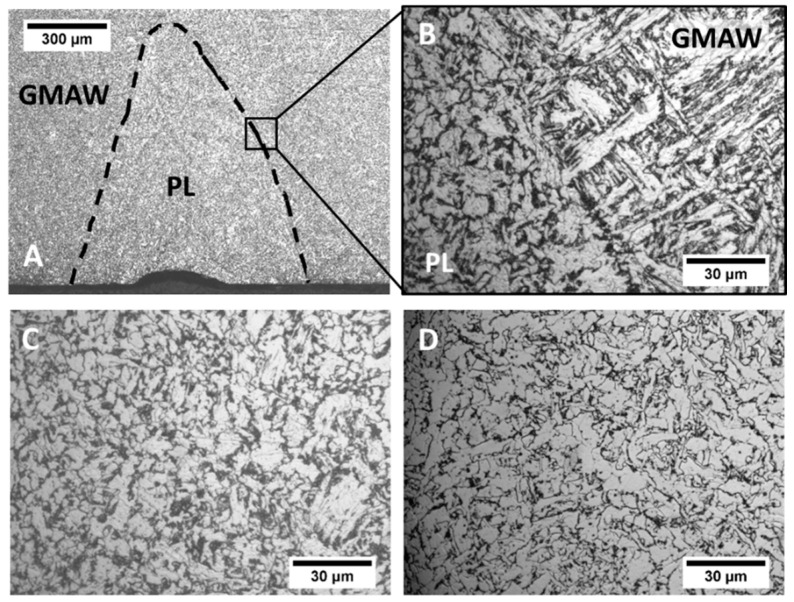
Microstructures of the root layer of the PL + GMAW condition showing in (**A**) the PL region, in (**B**) the processes interface, and in (**C**) the centre of the PL region. In (**D**), the centre of the root layer of the GMAW condition is shown.

The difference in the welding conditions was mainly in the bottom part of the weld seam. The PL beam generated a welding area with about 1 mm depth with a small root concavity measuring 53 µm in depth (Figure 5A). The welding area developed by the PL was thermally exposed by the following GMAW process, which modified its characteristics. In this sense, the interface between both processes was distinguished by the difference in the microstructure. The PL area showed tempered martensite with carbides agglomerates, whereas the GMAW area produced bainite with polygonal ferrite (Figure 5B). PL + GMAW and GMAW bottom regions presented a microstructure composed of ferrite with cementite agglomerates (Figure 5C,D). In the study performed by Ikram et al. [36], related to the influence of the gap distance with AH36 using ER-70S filler wire, the weld metal presented similar microstructures.

It is possible to determine the effect of the GMAW by comparing the FZ microstructure of the PL before and after its application (Figure 2 and Figure 5). The arc process thermally affected the laser area and provoked modifications in the microstructure. Notably, the martensite and bainite were transformed into a ferrite polygonal with carbides agglomerates and acicular ferrite. Martensite and bainite are associated with rapid cooling, and in the present study, the fast-cooling rate developed by the PL process was responsible for this transformation. Due to the subsequent heat exposition generated by the arc welding, this region was exposed to a tempering process, transforming the microstructure.

#### 3.2.2. Microhardness

**Figure 6:**
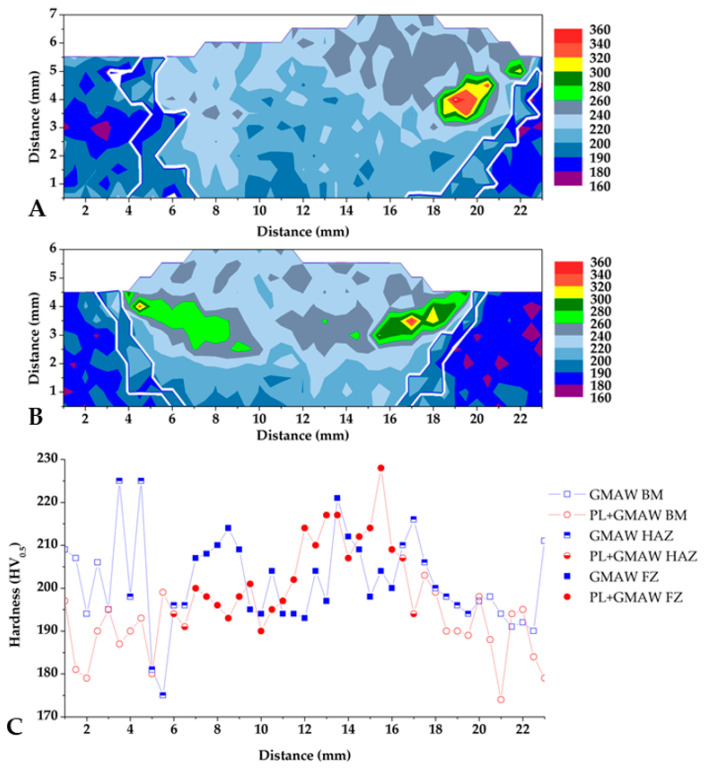
Microhardness mapping of GMAW (**A**), PL + GMAW (**B**), and the bottom-line profile (**C**). The white lines delimit the HAZ in (**A**,**B**).

Microhardness testing was performed and the results are displayed in Figure 6. The hardness distributions measured in all joint sections are displayed through mapping. The base material (extremities of each hardness mapping) presented the lowest hardness, with an average of 186 HV. The values measured correspond to the ferritic–pearlitic microstructure. As expected, the joint area presented an increase in values (centre of each mapping), and the additional welding pass of the arc condition caused a difference in the shape of the weld bead (as seen in the macrographs).

The upper half of both welding conditions indicated higher values, corresponding to the GMAW process incidence. This area displayed a microstructure composed of bainite, acicular ferrite, grain boundary ferrite, and Widmanstatten. The high values were associated with a higher concentration of finer and harder microstructure, such as bainite. In particular, the peak in the PL + GMAW and GMAW conditions were similar, 346 and 365 HV_0_._5_, respectively.

The heat-affected zone definition was performed by the visualization of the microstructure modification during the hardness testing. This procedure allowed its identification with a white line in the mappings. It is worth mentioning that the size of the HAZ is three times higher in GMAW than in PL + GMAW condition. The additional arc welding pass of the GMAW makes this zone much larger than in the PL + GMAW condition.

Regarding the behaviour of the welding roots, the line carried out at 0.5 mm from the bottom surfaces hardly indicated a significant difference between the processes in terms of values measured. The peak of the PL + GMAW was 228 HV, and 221 HV in the GMAW. The values of the PL condition showed the thermal effect of the arc process applied after the laser, reducing from approximately 500 HV to 228 HV.

Other investigations made with the GMAW process applied to the AH36 steel were performed using a similar filler wire (ER-70S). These studies indicated lower hardness values: in [36], the peak was 225 HV, whereas in [37] the highest number was 270 HV. The different hardness values may be associated with the different chemical compositions of the filler wires, as well as the process condition, which defines the microstructure developed by the heat input and the cooling rate applied.

#### 3.2.3. Charpy Impact Testing

**Figure 7:**
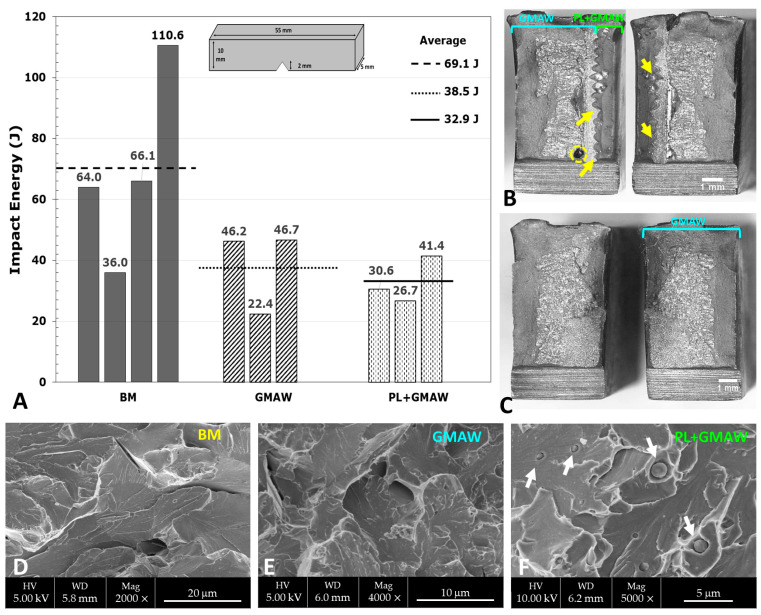
Results of the Charpy test at −50 °C (**A**) and the fracture surfaces of the PL + GMAW (**B**) and GMAW (**C**). SEM micrographs of the BM (**D**), GMAW (**E**), and PL + GMAW (**F**).

The toughness was measured in the ZF by the Charpy impact test, whose results are depicted in Figure 7. It indicated the welding processes effects in the average values, displaying 38.5 J in the GMAW and 32.9 J in the PL + GMAW. Both conditions achieved energy absorbed under the base material (69.1 J), representing around 55 and 47% of the toughness, respectively. Compared to the welding processes, a reduction of about 15% in toughness was noted, reflecting the influence of the PL process, even though these values are similar to those obtained by Yilmaz and Gunay [38] in a study performed in the AH36 steel welded with the submerged arc welding (SAW) process. The Charpy impact test was measured in [38] at −40 °C, providing values between 22.56 and 48.43 J. However, it is worth mentioning that, in the present investigation, the testing was carried out at −50 °C.

After the Charpy testing, the fracture surface morphology evaluation was carried out in all specimens using a stereo microscope and SEM. The analyses indicated that all fractographies had a central brittle area with shear in the outer zone. Particularly, the brittle area followed the interface between both welding processes in the PL + GMAW specimen that displayed the lowest energy value. It was possible to note the path through the sequence of the PL spots with a zigzag shape (arrows in Figure 7B). Additionally, this surface also displayed a void with around 0.5 mm of diameter in the GMAW zone Figure 7.

The evaluation of the fracture surface morphology carried out in SEM revealed a cleavage fracture with some intergranular facets in all conditions. The PL + GMAW specimen with the lowest energy value displayed reduced inclusions nucleated within the cleavage.

#### 3.2.4. Tensile Testing

**Figure 8:**
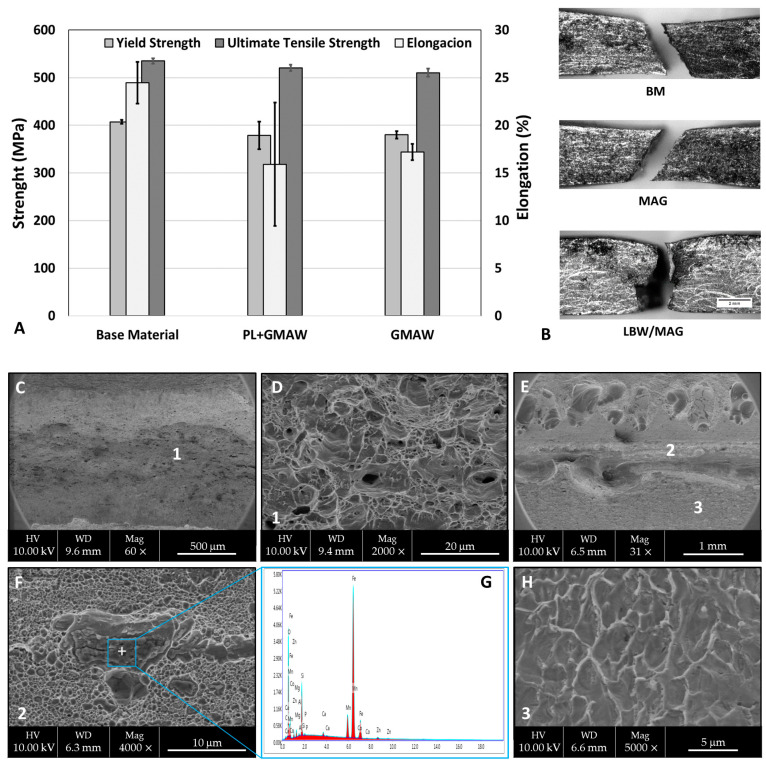
(**A**) Tensile properties of the base material and welded specimens. (**B**) Fractured specimens SEM images of fracture surface of the GMAW condition (**C**,**D**) and PL + GMAW condition (**E**,**F**,**H**). EDS spectrum of inclusion detected in PL + GMAW sample (**G**).

The strength performance of the welded specimens is shown in Figure 8. As observed, the tensile properties of the base material were higher than in both welding conditions. In terms of joining processes, a clear difference between the processes was not noted. The BM ultimate tensile strength (UTS) was 534.9 MPa, whereas the GMAW condition displayed 510.3 MPa and the PL + GMAW 520.4 MPa, corresponding to 97.5% and 95.4% BM. The higher value of elongation was measured in the BM at 25.5%, followed by 17.2% GMAW and 15.9% PL + GMAW. All samples had a fracture in the base material, except one specimen of PL + GMAW, which failed in the welding zone. This specimen was responsible for the higher value of deviation.

A deep evaluation of the fracture surface of the tensile test specimens was carried out in both welding conditions. The GMAW condition displayed a fully ductile morphology with a coalescence of microvoids. As the sample did not fracture in the weld zone, this characteristic corresponds to the base material. On the other hand, the surface of the PL + GMAW sample showed two different zones. One zone presented ductile fracture where a microvoid coalescence mechanism was observed. The dimples were nucleated at particles and the EDS analyses revealed that they were inclusions. Another zone was characterised as the quasi-cleavage that may be associated with the welding zone, as this specimen fracture in this region.

The tensile characteristics analysed are in accordance with another investigation using the same materials. The study performed by Ikram et al. [36] about the influence of the gap distance with AH36 and ER-70S filler wire indicated that all the welded samples were fractured at the fusion line, and the strength was below the AH36 parent material.

Based on these results, the difference between GMAW and PL + GMAW samples relies on the size of the welding zone and its bottom part. The higher number of arc welding passes of the arc condition generated a larger weld seam. In addition, the PL beam created a welding area of about 1 mm depth. The PL microstructure changed with the GMAW application due to the exposition of the thermal cycles generated by the following arc passes. In particular, the martensite and bainite observed in the PL root pass were transformed into polygonal ferrite with carbides agglomerates. In this sense, the main effect of the arc process was to modify the brittle microstructure developed by the PL, generating a tempering process. As a consequence, the hardness reduced significantly from about 500 to 230 HV. The values were below 350 HV, corresponding to the limit specified by some maritime classification societies [25,26]. Nevertheless, regarding the entire welding zone, the peaks of the PL + GMAW were slightly lower than the GMAW, 346 and 365 HV_0_._5_, respectively. The GMAW measurement was observed punctually, though there are above the industrial requirements.

In addition, the strength performances of both methods were very similar, where the ultimate tensile strength of the PL condition was 5% higher than the GMAW. Nevertheless, the values were not superior to the base material (up to 5% below), which is the standard requirement. Regarding the Charpy testing, the toughness indicated a reduction of 15% when the laser was applied.

Overall, the use of the PL as a root layer only demonstrated a significant difference in the mechanical properties in the toughness testing. The application of the following arc process introduced a new and slow thermal gradient, which was responsible for transforming the microstructure, hardness, and Charpy toughness, without reducing the strength performance.

According to these outcomes, in terms of metallurgical and mechanical results, further investigations should be carried out to evaluate the use of PL as a root pass before GMAW. As the only detrimental effect of the PL was observed in the toughness, other studies could be developed varying the process parameters or using equipment with higher capacities in order to achieve a deeper penetration in the PL zone.

## 4. Conclusions

This investigation dealt with the effect of the PL process applied as a root pass before the GMAW process in 5.5 mm thick AH36 steel. The main findings of this work are listed as follows:

- The weld seams produced with GMAW and PL + GMAW displayed similar macrostructure characteristics; however, the HAZ of the arc condition was larger. The depth of laser influence was about 1 mm.
- A significant influence of the GMAW process was observed in the PL root microstructure. The thermal cycle of the GMAW passes transformed the martensite and bainite into polygonal ferrite with carbides agglomerates.
- In terms of mechanical properties, both welding conditions displayed similar tensile strength performance and hardness results, though the Charpy impact values of the PL + GMAW were around 15% lower than the GMAW.
- From this study, it can be stated that GMAW welding passes smoothed the microstructure of previous root laser welding processes, reducing, therefore, the hardness of the PL zone.

## Figures and Tables

**Table 1:** Chemical composition (wt%) of the AH36 steel and filler wire.

Element Composition (max.)	C	Mn	Si	P	S	Cr	Al	V	Nb	Ni
AH36 [28]	0.18	0.90–1.60	0.10–0.50	0.035	0.035	0.20	0.015	0.05–0.10	0.05–0.05	-
Filler Wire [29]	0.12	1.75	0.9	0.03	0.03	0.20	-	0.08	-	0.5

**Table 2:** Welding parameters used in PL and GMAW processes.

**Pulse Laser Parameters**		
**Characteristics**	**Values**	
Pulse duration	14 ms	
Peak power	5.04 kW	
Spot diameter	1.2 mm	
Frequency	0.5 Hz	
Focus position	Upper surface	
Shielding gas	Argon, 15 L/min, 180 bars, 45°	
Frequency	0.5 Hz	
Overlap	0.5 mm	
Welding speed	0.25 mm/s	
**GMAW parameters**		
**Condition**	**PL + GMAW**	**GMAW**
Run	1/2/3	1/2/3/4
Amplitude (A)	190/189/192	186/186/187/191
Voltage (V)	20.8/20.6/20.6	20.8/20.9/19.9/20.2
Weld speed(cm/min)	52.6/34.3/37.7	36.3/35.7/33.3/34.4
Heat Input(kJ/mm)	0.36/0.54/0.5	0.51/0.50/0.53/0.54
Interpass temp. (°C)	22.5/85.6/88.2	32.0/86.0/84.4/85.1

## Data Availability

Not applicable.
